# ChatGPT in Medical Education and Research: A Boon or a Bane?

**DOI:** 10.7759/cureus.44316

**Published:** 2023-08-29

**Authors:** Madhan Jeyaraman, Shanmuga Priya K, Naveen Jeyaraman, Arulkumar Nallakumarasamy, Sankalp Yadav, Suresh K Bondili

**Affiliations:** 1 Orthopaedics, South Texas Orthopaedic Research Institute (STORI), Laredo, USA; 2 Orthopaedics, ACS Medical College and Hospital, Dr MGR Educational and Research Institute, Chennai, IND; 3 Pulmonology, Faculty of Medicine, Sri Lalithambigai Medical College and Hospital, Dr MGR Educational and Research Institute, Chennai, IND; 4 Medicine, Shri Madan Lal Khurana Chest Clinic, New Delhi, IND; 5 Medical Oncology, Kauvery Hospital, Chennai, IND

**Keywords:** research, medical education, open ai, chatgpt, artificial intelligence, ai & robotics in healthcare

## Abstract

Science fiction literature and films no longer focus on artificial intelligence. In contrast to all other aspects of life, medical education and clinical patient care have been progressing slowly. Recently, a lot of text from the internet was used to construct and train chatbots, especially ChatGPT. The language model ChatGPT, created by OpenAI, has emerged as a useful resource for medical research and education. It has proven to be a useful tool for researchers, students, and medical professionals because of its capacity to produce human-like answers to challenging medical queries. However, using ChatGPT also has significant drawbacks. The possibility of erroneous or biased information being spread, which could have negative effects on patient care, is one of the key worries. Moreover, the overreliance on technology in medical education could also lead to a decline in critical thinking and clinical decision-making skills. Overall, ChatGPT has the potential to be a boon to medical education and research, but its use must be accompanied by caution and critical evaluation.

## Introduction and background

Due to the rapid advancement of scientific knowledge and technology, experts now frequently use new artificial intelligence (AI) models for convenience and quick access to necessities. Computers that read user input and deliver responses are used by Google and Meta to create complex language model tools [1]. To develop predictions and combine words in meaningful ways, a lot of data and computing methods are used. Similar to this, in November 2022, the artificial intelligence company "Open AI" unveiled the brand-new trending bot "ChatGPT." Millions of users and investors have invested in this bot, and scientists predict that it will eventually replace people. By creating a user interface that allows the general public to interact with it directly, Open AI made a revolutionary choice [1]. Since its launch, ChatGPT has undergone extensive testing across a range of disciplines to ensure that it operates naturally and conversationally. Technology breakthroughs and the creation of AI technologies like language models like ChatGPT have considerably helped medical education and research. However, whether these technological advancements are a boon or a bane depends on how they are utilized. When replying to medical inquiries or providing differential diagnoses for various symptoms, it has been utilized in health care to provide medical aid and information [2].

Big language models can help with clinical decision-making and medical education. According to new research by Kung et al., ChatGPT performed at or near the passing level for all three United States Medical Licensure Exams (USMLE) [3]. How much ChatGPT can impact medical research is a crucial question. Following Dr. Biswas, this software can revolutionize medical writing by speeding up the process and saving time. It can gather data, help with literature searches, and produce a preliminary document that the medical writer can then refine [4]. It can be quite advantageous for the medical sector. It can aid in the efficient diagnosis of patients by providing a summary of their vast medical records based on their family history, lab results, and symptoms. Because ChatGPT lacks critical thinking and provides information redundantly and arbitrarily, many scientists and journals are opposed to it [5].

According to numerous education experts, students taking communication and philosophy courses frequently use the easily detectable exam cheating tool ChatGPT. A growing worry is that people may eventually lose the ability to come up with fresh ideas and won't be able to provide sound justifications for their positions [5]. Similar to this, the accountability of the content of the bot is a problem when employing ChatGPT in scientific articles. That raises ethical questions, medical law issues, copyright issues, a dearth of original thought and reasoning, methodological biases, and content inaccuracies [6]. When using ChatGPT for research, it might be simple to create a paper's content using the data from search engines. However, as previously mentioned, it was unable to conduct a thorough literature review or evaluate and discuss articles [7]. The only observable result was a rephrased language that depends on the precise instructions given to the bot and is not entirely free of plagiarism. According to Mayo Clinic doctors Thomas Davenport and Nitin Mittal, ChatGPT can be abused innumerably. It might eventually prevent the human mind from doing even the most basic tasks [8,9]. Also, ChatGPT's current training data, which is updated through 2021, was mentioned as one of the main barriers to adoption by John Halamka (President of the Mayo Clinic platform) and Paul Cerrato (Senior Research Analyst and Communications Specialist, Mayo Clinic platform) [10]. Because medical literature is updated frequently, there is growing concern that papers created using ChatGPT may lack clinical reasoning and thinking.

ChatGPT can be a boon for medical education and research. It has the potential to provide instant access to a vast amount of medical information and can assist medical students and researchers in analyzing complex medical data. Additionally, ChatGPT can provide personalized learning experiences for medical students by tailoring information to their learning styles and preferences. ChatGPT also poses some challenges and limitations that can be considered a bane. While ChatGPT can provide vast amounts of information, it may not always be accurate or reliable. The responses produced by ChatGPT are also based on patterns and trends in the data it has been trained on, so they might not always offer a thorough or nuanced knowledge of a specific medical idea or situation. Consequently, it is crucial to make sure that medical students and researchers are aware of ChatGPT's limitations and use it in addition to rather than as a substitute for conventional learning and research techniques. All things considered, ChatGPT has the potential to be a useful tool in medical research and education, but it must be used carefully and with awareness of its limitations. This review article focuses on whether ChatGPT in medical education and research is a boon or a bane.

## Review

Here are a few basic similes that could be used to describe ChatGPT:

· ChatGPT is like a virtual assistant, always ready to help you with your language-related tasks.

· ChatGPT is like a language genie, granting your wishes for information and conversation in the blink of an eye.

· ChatGPT is like a language chameleon, adapting to any language and communication style to help you communicate effectively.

· ChatGPT is like a language atlas, mapping out the vast landscape of human language and knowledge to help you navigate it more efficiently.

· ChatGPT is like a language encyclopedia, containing a wealth of information and knowledge on a wide range of topics to help you learn and communicate better.

Can ChatGPT influence health care?

ChatGPT, a cutting-edge language model created by Open AI, has the potential to fundamentally alter how medical information is handled and communicated in the disciplines of clinical and translational medicine [11].

· Access to current data: ChatGPT can access a huge amount of medical data and respond to clinical queries in real time with precise answers.

· Increased patient engagement: Patients can track their health information with ChatGPT and obtain answers to their questions regarding medicine straightforwardly and rapidly.

· The lessened workload for medical professionals: ChatGPT can assist in lessening the administrative strain on medical professionals, allowing them to concentrate more on patient care.

· Accuracy will certainly increase as more data are gathered and examined for ChatGPT.

· ChatGPT may be able to communicate with electronic health records (EHRs), facilitating a more fluid exchange of data between patients and healthcare professionals.

· Customized medicine: Based on unique patient information and health histories, ChatGPT has the potential to offer individualized medical recommendations.

· To find new medication targets and create individualized treatment programs for patients, enormous volumes of health data, including patient genomes, are analyzed.

· Clinical studies that are optimized to find the most promising participants.

ChatGPT and medical education

Technology breakthroughs are advancing medical education, and AI like ChatGPT can serve several beneficial functions, which are as follows [12]:

· Automatic scoring: The clarity, vocabulary, grammar, and sentence structure of student essays and papers can be evaluated using ChatGPT. Teachers and other educators who routinely deal with the enormous effort required for grading a large number of assignments will find this feature to be of particular use.

· Teaching assistance [13,14]: ChatGPT can be used to create exercises, tests, and scenarios that will help with practice and assessment in the classroom. Its capacity to produce translations, justifications, and summaries can also be used to simplify for students difficult academic material.

· Personalized learning [15]: ChatGPT can be used to create virtual tutors or assistants who can assist students with their homework and ask questions. Teachers may find it challenging to create individualized study plans and learning materials for each student in a classroom setting that take into account people's diverse learning styles and abilities.

· Research assistance [16]: ChatGPT can be used to help students with their academic work by responding to their questions and writing summaries. Moreover, it can be used to create outlines, bibliographies, and other types of research tools. ChatGPT's capacity to aid in the creation of outlines, as well as with literature reviews and data analysis, can make medical research simpler. Also, it can be used to summarize pertinent publications and highlight crucial discoveries, assisting medical researchers in making sense of a large amount of material available online.

· Fast access to information [17]: Using ChatGPT, you may quickly provide accurate and current information about medical topics. This could cover a wide range of issues, including illnesses and how they are treated as well as medical procedures. Students and medical professionals who require immediate access to information or clarification on a topic could find this useful.

· Creating case scenarios [18]: Medical students can hone and improve their diagnostic and treatment planning skills by using ChatGPT to create case studies and scenarios. This could aid students in developing their aptitude for clinical thinking as well as preparing them for situations that may arise in actual clinical settings.

· Language translation [19]: By utilizing ChatGPT's language translation tools, medical professionals and educators may communicate with patients from various linguistic backgrounds and deliver the finest medical treatment.

ChatGPT and medical research

One benefit of employing ChatGPT in research may be its capacity to analyze vast amounts of data rapidly and accurately, including patient records, medical reports, scientific journals, and medical reports, all of which can offer unique insights into the causes, symptoms, and treatment possibilities. According to ChatGPT, natural language processing techniques are utilized to extract crucial information from the texts and present it in a structured way. Another novel application of ChatGPT is to assist researchers in the creation of novel theories. ChatGPT may be able to come up with fresh suggestions for additional studies by analyzing the current literature and identifying knowledge gaps [20]. By examining patient records and finding common trends, ChatGPT may be able to help with the creation of clinical decision support systems. Authors have already acknowledged ChatGPT as a co-author by utilizing the AI technology that ChatGPT enabled [21,22]. However, not all scientists and authors agree, as Thorp recently stated that “ChatGPT is fun, but not an author” [6].

It is true that because the authors did not produce the content themselves, ChatGPT may be used to produce articles that may be considered to be plagiarized. Another potentially hazardous application of ChatGPT is when researchers use it to create material that is eerily similar to passages or sections of previously published works. Moreover, ChatGPT may generate language that is almost the same as that found in previously published studies. This capability can be exploited to fudge study results or mislead readers and researchers. The question of whether human reviewers could discern between authentic scientific abstracts and ChatGPT-written AI-generated abstracts was examined in a recent study that was posted on a preprint server [13,23]. Blinded reviewers acknowledged having trouble telling the difference between abstracts produced by humans and those produced by AI. In fact, in 32% of the abstracts produced by the AI bot, ChatGPT was successful in deceiving blinded reviewers. Emerging methodologies designed to distinguish between human-authored content and generative AI-generated content, such as the Japanese stylometric analysis elucidated by Zaitsu and Jin, warrant prudent application due to their varying levels of accuracy across distinct scenarios [24]. The generated writings could also be erroneous, biased, and lack comprehension of the subtleties of medical science(s) and language, in addition to the issue with plagiarism (ChatGPT is trained on a large dataset of text but may not have enough information about a specific case). Certain topics don't have as many articles; therefore, the AI bot can undervalue their significance or distinctiveness. The AI bot might thus prevent researchers from evaluating the available data based on quality and instead restrict them to a more broad perspective. It is crucial to advise future ChatGPT users to use the tool responsibly and to always properly reference any materials utilized to avoid the problems highlighted above.

Advantages of ChatGPT

· Improved learning: ChatGPT can provide medical students with instant access to a vast amount of medical knowledge, which can help them learn quickly and efficiently. Giving students immediate feedback on their comprehension of the subject matter can also improve the learning experience.

· Time-saving [25]: With ChatGPT, medical professionals and researchers can save time in researching, writing papers, and studying as the information can be accessed quickly.

· Personalized learning: ChatGPT can provide personalized learning experiences, tailoring the content to the specific needs of each learner.

· Better medical diagnosis: With ChatGPT's ability to process large amounts of medical data, it can help medical professionals make better diagnoses and provide better treatment options for their patients.

Disadvantages of ChatGPT

· Inaccurate or misleading information may hurt patients, and ChatGPT is not a replacement for professional medical care.

· Technology dependence: ChatGPT might be used as a crutch by medical professionals, limiting their capacity to diagnose and treat patients without the use of technology.

· Limited to existing data: ChatGPT can only provide information based on the data that has been previously input into its system. This means that if there is a gap in knowledge or a lack of data, ChatGPT will not be able to provide accurate information.

· Lack of critical thinking [26]: ChatGPT is a machine and cannot think critically like a human, meaning it cannot interpret or analyze medical information beyond what is programmed into its system. It cannot provide judgment or discernment required for ethical or legal aspects of medical practice.

· Data privacy [27]: Data privacy and security are essential in medical research and practice. The use of ChatGPT may require the sharing of medical data, which can pose a risk of data breaches and privacy violations.

· ChatGPT might replace conventional learning tools such as libraries and tutors [28].

· Bias in the training data: The quality of ChatGPT depends on the training data. The model could reinforce a bias if the training set of data is skewed.

Limitations of ChatGPT in medical education and research

Although the text produced by ChatGPT, an AI service, may be remarkably similar to content written by humans, it does have errors, just like any other statistical model. Its main faults include a deficiency in human-like comprehension and a lack of data input after 2021. This could allow it to ignore the context of the prompt and lead to the creation of pointless content or genuinely generic ideas and notions [28,29].

Recently, peer-reviewed publications using ChatGPT as an author were published [30]. Due to the aforementioned limitations, the World Association of Medical Editors (WAME) has suggested including ChatGPT with the authors [31,32]. The correctness and cultural appropriateness of the translations were not the focus of this investigation. The effectiveness of apps in healthcare settings may be impacted by the appropriateness of translated words for the context, the syntax of translated phrases (such as word order and grammar), and the ability to recognize different accents and dialects (when using free voice input). Studies in the past have shown that Google Translate's translation of non-Western languages is less accurate [33-35].

Remarkably, the first query about the possible impact of ChatGPT on clinical and translational medicine nearly seems to have a resolution [33]. But, given the widespread debate that has started around ChatGPT and its effect on health care, several issues need to be addressed. AI in medicine has indisputable advantages and benefits that have already impacted every element of the industry, from research to clinical applications [11]. AI-based algorithms are employed everywhere, from data-driven preclinical research to medical decision assistance in everyday clinical practice. For instance, standardized medical procedures in line with good clinical practice (GCP) already include pattern recognition, pre-interpretation of physiological and biophysical data like ECG, EEG, and EMG, as well as medical image interpretation for patient management and diagnosis [36].

However, ChatGPT's warnings about difficulties and risks inevitably prompt a lot of inquiries, including pertinent questions about medical ethics [37,38], data security and privacy, as well as inaccurate or deceptive information that could harm patients. All other things being equal, patients come across as certain and ready for the doctor's interview, especially when it comes to crucial clinical data such as disease-specific symptoms and medical history. Unfortunately, this frequently does not serve the attending physician's best interests and might result in misunderstandings. Also, if ChatGPT is utilized without permission, its variable accuracy can cause issues in the educational setting. This instrument has already been prohibited at certain universities. Medical students and trainees may be negatively impacted by ChatGPT, leading them to incorrectly interpret medical information or even make clinical judgments without utilizing their judgment. So, before it can be utilized, for instance, for applications that accompany diagnosis and therapy, the ChatGPT algorithm needs to be trained and verified on relevant, evidence-based knowledge bases. So, before it can be utilized, for instance, for applications that accompany diagnosis and therapy, the ChatGPT algorithm needs to be trained and verified on relevant, evidence-based knowledge bases [39]. By facilitating access to current information, enhancing patient engagement, and lessening the burden on the medical staff, ChatGPT has the potential to make a substantial impact on the disciplines of clinical and translational medicine [40].

ChatGPT has the potential to have a significant impact on the fields of clinical and translational medicine by simplifying access to up-to-date information, improving patient participation, and reducing the workload on the medical staff. To ensure that ChatGPT is used securely and successfully, further study and development are required. Yet, some difficulties and dangers must be taken into account and avoided. It is used as the desire and moral duty to enhance the patient's quality of life. As an AI language model, ChatGPT has the potential to play a significant role in medical research in the future. Here are some ways that ChatGPT can be used:

· Data analysis: ChatGPT can be used to analyze large sets of medical data, such as electronic health records, medical images, and patient outcomes. It can identify patterns and trends that may be missed by human analysts, which can be useful in identifying potential risk factors and predicting health outcomes.

· Literature review: ChatGPT can assist in conducting a comprehensive literature review for a given research question, by analyzing a vast amount of medical literature and providing a summary of relevant information.

· Decision support: ChatGPT can be used to assist healthcare providers in making treatment decisions by analyzing patient data and providing personalized treatment recommendations based on the latest medical evidence.

· Patient engagement: ChatGPT can be used to provide patients with accurate and reliable information about their health conditions, treatments, and preventive measures. It can also help patients make informed decisions about their health by answering their questions in real time.

· Drug discovery: ChatGPT can be used to predict drug candidates for a given target by analyzing the chemical and biological properties of thousands of compounds. This can help researchers save time and effort in identifying potential drugs for a given target.

· Clinical trial design: ChatGPT can assist in designing clinical trials by identifying potential inclusion and exclusion criteria, selecting appropriate endpoints, and analyzing the potential risks and benefits of the study.

However, it is important to note that ChatGPT is an AI language model and not a substitute for medical professionals. It should always be used in conjunction with expert medical advice and clinical judgment. Additionally, as with any AI technology, there are potential risks and ethical concerns that need to be carefully considered and addressed, such as data privacy, bias, and accountability. ChatGPT is an AI language model that has the potential to assist humans in many tasks, including in the field of medicine and medical research. However, it is important to note that AI technologies like ChatGPT are not designed to replace humans in these fields, but rather to augment and assist human capabilities.

While AI technologies like ChatGPT can perform some tasks more efficiently than humans, they are not capable of replacing human expertise, judgment, and empathy. Many aspects of healthcare and medical research require human skills, such as diagnosing complex medical conditions, interpreting medical imaging, and communicating with patients about their care. Furthermore, the use of AI technologies like ChatGPT in health care and medical research must be carefully regulated and monitored to ensure that they do not cause harm or perpetuate bias. Ethical considerations, such as data privacy, transparency, and accountability, must also be taken into account when using AI technologies in these fields.

Therefore, it is unlikely that ChatGPT or any other AI technology will completely replace humans in the field of medicine and medical research. Rather, these technologies will be used to assist and augment human capabilities, leading to more accurate diagnoses, personalized treatments, and better patient outcomes. ChatGPT is not a means of war, even after the chatbot. As an AI language model, ChatGPT is designed to assist humans in various tasks such as language translation, text summarization, and conversation generation. Its intended purpose is to improve the efficiency and accuracy of human communication and information processing, not to be used as a weapon of war or to cause harm to others.

While it is true that AI technologies, including chatbots, have been used in various military applications, such as autonomous weapons and surveillance systems, it is important to note that the development and use of such technologies are subject to strict ethical and legal regulations. The use of AI technologies in warfare must comply with international humanitarian law and the principles of proportionality, distinction, and military necessity. Furthermore, the development and use of AI technologies, including ChatGPT, must adhere to ethical and moral considerations, including transparency, accountability, and the protection of human rights. The responsible development and use of AI technologies can bring significant benefits to society, but it is important to ensure that they are used for beneficial purposes and do not cause harm to others.

## Conclusions

Utilizing ChatGPT in the realms of clinical management, medical research, and education holds immense potential, but it should not supplant human expertise, given the inherent limitations of AI. As advancements in information technology, machine learning, and AI rapidly evolve, it is imperative to approach their integration into the medical sector with both eagerness and caution. ChatGPT can streamline the acquisition of up-to-date information, bolster patient engagement, and alleviate some demands on medical professionals, especially within clinical and translational medicine. For the ethical and effective employment of ChatGPT, it is crucial to continuously assess its applications, make necessary adaptations, and invest in thorough research. This will safeguard its use while maximizing the benefits it offers to the medical community.
